# Cerebrospinal Fluid Findings in 541 Patients With Clinically Isolated Syndrome and Multiple Sclerosis: A Monocentric Study

**DOI:** 10.3389/fimmu.2021.675307

**Published:** 2021-06-17

**Authors:** Klaus Berek, Gabriel Bsteh, Michael Auer, Franziska Di Pauli, Anne Zinganell, Thomas Berger, Florian Deisenhammer, Harald Hegen

**Affiliations:** ^1^ Department of Neurology, Medical University of Innsbruck, Innsbruck, Austria; ^2^ Department of Neurology, Medical University of Vienna, Vienna, Austria

**Keywords:** white blood cells, cytology, immunoglobulin synthesis, albumin quotient, total protein, diagnosis, multiple sclerosis, cerebrospinal fluid

## Abstract

**Background:**

Reports on typical routine cerebrospinal fluid (CSF) findings are outdated owing to novel reference limits (RL) and revised diagnostic criteria of Multiple Sclerosis (MS).

**Objective:**

To assess routine CSF parameters in MS patients and the frequency of pathologic findings by applying novel RL.

**Methods:**

CSF white blood cells (WBC), CSF total protein (CSF-TP), CSF/serum albumin quotient (Q_alb_), intrathecal synthesis of immunoglobulins (Ig) A, M and G, oligoclonal IgG bands (OCB) were determined in patients with clinically isolated syndrome (CIS) and MS.

**Results:**

Of 541 patients 54% showed CSF pleocytosis with a WBC count up to 40/μl. CSF cytology revealed lymphocytes, monocytes and neutrophils in 99%, 41% and 9% of patients. CSF-TP and Q_alb_ were increased in 19% and 7% applying age-corrected RL as opposed to 34% and 26% with conventional RL. Quantitative intrathecal IgG, IgA and IgM synthesis were present in 65%, 14% and 21%; OCB in 95% of patients. WBC were higher in relapsing than progressive MS and predicted, together with monocytes, the conversion from CIS to clinically definite MS. Intrathecal IgG fraction was highest in secondary progressive MS.

**Conclusions:**

CSF profile in MS varies across disease courses. Blood-CSF-barrier dysfunction and intrathecal IgA/IgM synthesis are less frequent when the novel RL are applied.

## Introduction

Diagnosis of multiple sclerosis (MS) requires the combination of clinical signs and symptoms with paraclinical findings obtained by magnetic resonance imaging (MRI) and cerebrospinal fluid (CSF) analysis (1). Evidence of intrathecal immunoglobulin (Ig) G synthesis in the CSF measured by oligoclonal bands (OCB), although not specific for MS, increases diagnostic certainty in the appropriate clinical setting (2) and substitutes for dissemination in time (DIT) according to current diagnostic criteria (1). Conversely, CSF findings atypical of MS suggest other diseases. To distinguish between MS and other causes of central nervous system inflammation, e.g. vasculitis, chronic infection or acquired demyelinating disorders other than MS, the full spectrum of routine CSF parameters is applied that includes white blood cell (WBC) count, cellular differentiation, albumin quotient (Q_alb_) and intrathecal Ig synthesis (3).

While the typical CSF findings in patients with MS have been previously reported (4, 5) and frequently cited in clinical practice guidelines (3, 6, 7), these studies already date back to last century and the majority of them were largely confined to the intrathecal Ig synthesis (8–10). Furthermore, some findings, e.g. a WBC count <50/μl (3), have not been re-evaluated in the view of the revised McDonald criteria (1), and the reported frequency of CSF abnormalities, e.g. increased Q_alb_ as indicator for the blood-CSF-barrier function, were based on reference limits that are outdated nowadays (11, 12).

The objective of the present study was to describe routine CSF parameters in patients with MS, to assess differences with regard to the disease course (e.g. relapsing *versus* progressive course) and to assess the frequency of pathological findings by applying novel reference limits (RL).

## Methods

**Figure 1:**
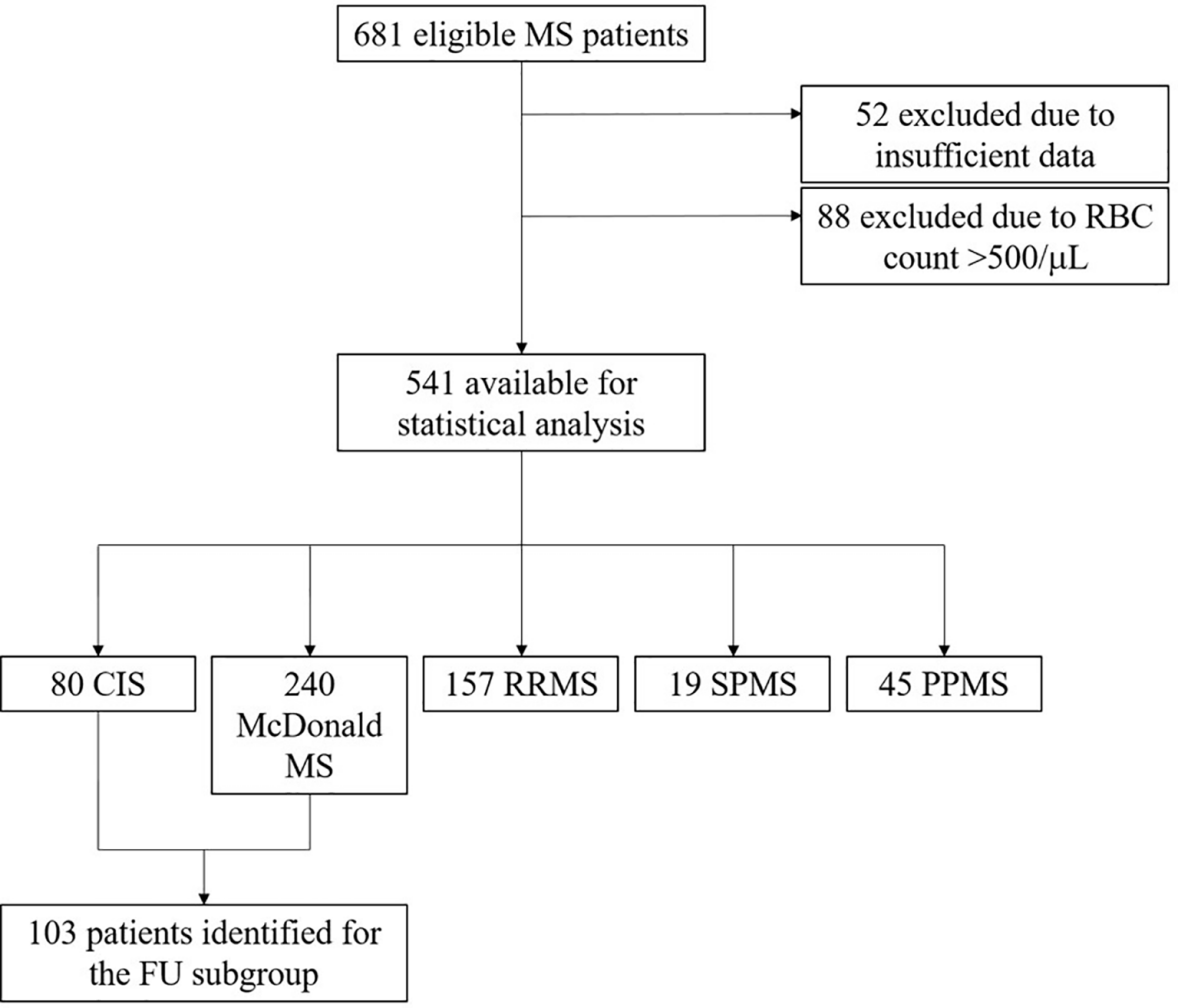
Flowchart of patients included in the study. CIS, clinically isolated syndrome; FU, follow-up; PPMS, primary progressive MS; RBC, red blood cell; RRMS, relapsing remitting MS; SPMS, secondary progressive MS.

Patients at the MS clinic of the Department of Neurology of the Medical University of Innsbruck who received the diagnosis of clinically isolated syndrome (CIS) or MS according to the 2017 revised McDonald criteria (1) and who had CSF analysis centrally performed at the Neuroimmunology Laboratory of Medical University of Innsbruck between January 2002 and July 2019 were eligible for inclusion into the study. This resulted in a total of 681 patients. Those with insufficient clinical data, e.g. to define MS disease course (n=52), or with bloody CSF, i.e. showing a red blood cell (RBC) count >500/μL (13) (n=88), were excluded. Finally, a total of 541 patients were available for statistical analysis (**Figure 1**).

Patients were categorized into following groups: CIS, McDonald MS, relapsing-remitting MS (RRMS), secondary-progressive MS (SPMS) and primary progressive MS (PPMS) applying the 2017 revised McDonald criteria (1) and the definition of disease courses by Lublin and Reingold (14), both at the time of lumbar puncture (LP) (baseline) and at follow-up (FU). Patients with McDonald MS had one clinical attack and fulfilled McDonald criteria (1) by paraclinical findings (MRI and/or CSF findings). In contrast, CIS patients had one clinical attack but did not fulfil diagnostic criteria for MS. RRMS patients fulfilled McDonald criteria (1) and experienced at least two relapses. Dissemination in space (DIS) in these patients was demonstrated either clinically by relapses involving different function systems, or by means of MRI (1).

Furthermore, patients with a first clinical attack at baseline were stratified based on their initial MRI findings into following groups: only DIS, only DIT, DIS and DIT, no DIS and no DIT. The definition of DIS was fulfilled by one or more T2-hyperintense lesions characteristic of MS in two or more of four areas of the CNS: periventricular, cortical or juxtacortical, infratentorial brain regions, and the spinal cord. DIT was demonstrated by the simultaneous presence of gadolinium-enhancing and non-enhancing lesions (1).

A subgroup of these patients, who were followed for at least one year, were eligible for stratification according to the occurrence of further relapse. MRI findings were analysed by two blinded independent investigators.

### Laboratory Assays and Sample Collection

CSF samples were collected by standard LP for routine diagnostic purposes. Blood samples were withdrawn simultaneously by peripheral venous puncture. Serum was isolated from blood by centrifugation after the blood samples were allowed to clot for ≥30 minutes.

The routine parameters comprised CSF RBC count, CSF WBC count, CSF cellular differentiation, CSF total protein (CSF-TP) as well as albumin, IgG, IgA and IgM and oligoclonal IgG bands (OCB) in CSF and serum.

CSF WBC and RBC were counted in a Fuchs-Rosenthal chamber, which has a volume of 3.2 μL (6). Division by 3.2 allowed reporting of cell counts per μL according to the International System of units (SI). Cellular differentiation in CSF was done by microscopy. Therefore, cytological preparation of CSF was made according to the method reported by Lehmitz et al. with minimal modifications (15). Briefly, 200 μl of undiluted, unconcentrated CSF was centrifuged on a glass slide, air-dried and subsequently stained using May-Grünwald & Giemsa staining (16). The different leukocyte subtypes were grouped into following categories: lymphocytes/plasma cells, monocytes/macrophages, neutrophils, eosinophils and basophils. Furthermore, the presence of “shadow cells” was assessed. Shadow cells were defined to be of pale, eosinophilic appearance, with (nearly) disappeared nuclear structures but with roughly maintaining the cytoplasmic silhouette (17).

CSF-TP was determined by spectrophotometry after incubation of centrifuged CSF with 3% trichloroacetic acid (18). Albumin, IgG, IgA and IgM were measured by nephelometry (Beckman Coulter GmbH, Brea, CA, USA) as previously reported (12). OCB were detected by isoelectric focusing and subsequent immunoblotting using IgG-specific antibody staining as previously described (19). OCB pattern were evaluated by experienced raters and classified as negative (pattern I, IV and V) or positive (pattern II, III) (3).

### Calculation of Various CSF Parameters and Their Reference Limits

The albumin quotient (Q_alb_) as an established marker of the blood-CSF-barrier function was calculated as the ratio of CSF albumin/serum albumin (6). Frequency of elevated Q_alb_ levels was assessed by applying the conventional upper reference limit (cURL) (20), Eq. (1), and a novel age-dependent URL (aURL) (21), Eq. (2).

Eq. (1) $$Q_{alb\;lim}=\frac{age}{15}+4$$

Eq. (2) $$Q_{alb\;lim}=0.0435\;\times \;age+7.9249$$

Frequency of increased CSF-TP concentrations was assessed using the cURL of 0.45 g/L (6), Eq. (3), and the aURL (22), Eq. (4).

Eq. (3) $$CSF\;TP_{lim}(g/l)\;=0.45$$

Eq. (4) $$CSF\;TP_{lim}(g/l)\;=\;0.124+0.0284\times age-7.08\times 10^{-4}\times age^{2}+8.23\times 10^{-6\;}\times age^{3}-3.35\times \;10^{-8}\;age^{4}$$

Intrathecal synthesis of IgG, IgM and IgA were determined by the Reiber formula (23), Eq. (5), and the Auer & Hegen formula (12), Eq. (6).

Eq. (5) $$Q_{lim}(IgX)=\frac{a}{b}\;\;\sqrt{Q_{alb}2+b^{2}\;}–\;c$$

Eq. (6) $$Q_{lim}(IgX)=\;a\times Qalb^{b}$$

Both formulae provide an URL that allows the determination of intrathecally produced Ig. Different constants *a*, *b*, and *c* have to be applied depending on the Ig isotype. The previously determined constants for the Reiber formulae (23) and Auer & Hegen formulae (12) are provided in the **Supplementary Methods**.

After *Q_lim_(IgX)* has been calculated, the relative intrathecal Ig fraction (IF) is calculated according to following formula:

$$Ig_{IF}(\%)=\left(1-\frac{Q_{lim}(IgX)}{IgX_{CSF}/IgX_{Serum}}\right)\;\times \;100$$

### Statistics

Statistical analysis was performed by SPSS 26.0 (SPSS Inc., Chicago, IL, USA). Distribution of data was assessed by Kolmogorov-Smirnov test. Non-parametric data were displayed as median and interquartile range (IQR) or 5^th^-95^th^ percentile, as appropriate. Categorical variables were reported as frequency and percentage. Spearman coefficient was used for correlation analysis. For group comparisons, Mann-Whitney U, Kruskal-Wallis test and Pearson Chi-squared test were applied. Multivariate regression analyses were performed including age, sex, disease duration and – depending on the precise research question – further covariables that differed statistically significantly between the respective patient groups by univariate comparisons, e.g. disease course. P-values <0.05 were considered statistically significant. P values marked as p* were corrected for multiple comparisons by Bonferroni.

### Ethics

We adhered to the guidelines of the declaration of Helsinki as well as the instructions of the Austrian Data Safety Authority (www.ris.bka.gv.at., 2018) by anonymizing patients’ data. In accordance with Austria’s national regulations for retrospective analyses of already existing data obtained for routine diagnostic procedures, no ethical committee’s vote is needed.

## Results

A total of 541 patients with a female predominance of 70% and a median age of 34 years at the time of LP were included into the study. In these patients, CSF analysis was performed a median of 30 minutes (IQR 20-50) after sample withdrawal. Eighty patients had the diagnosis CIS, 240 McDonald MS, 157 RRMS, 19 SPMS and 45 PPMS at the time of LP. Of 320 patients with a first clinical attack, 75 (23%) showed DIS only, 21 (7%) DIT only and 168 (53%) both DIS and DIT on brain MRI. A subgroup of 103 patients, other than those fulfilling DIS and DIT on MRI, were followed for median 9 years (IQR 5-12) for the occurrence of a second clinical attack. Details on demographics and main clinical characteristics of patients are shown in **Tables S1–S3**.

### CSF White Blood Cells

Overall, 291 (54%) of 541 patients showed an elevated WBC count ≥5/μl. In those patients with CSF pleocytosis, WBC count had a median of 10/μl reaching an upper limit of 40/μl (95^th^ percentile); the maximum WBC count was 65/μl. Visual inspection of CSF cytology identified cells in 403 (75%) of cases. Almost all patients (99%) showed lymphocytes, 41% monocytes and 9% neutrophils, while eosinophils and basophils were not detected (**Table 1A**).

**Table 1A:** Cerebrospinal fluid findings in multiple sclerosis and according to different disease courses: Cells and blood-CSF-barrier.

	Whole cohort		CIS		McDonalds MS		RRMS		SPMS		PPMS		P value
	n		n		n		n		n		n		
**Cells and Cytology**													
RBC (/μl)^1^	541	0 (0-120)	80	0 (0-256)	240	0 (0-59)	157	0 (0-134)	19	0 (0-201)	45	0 (0-49)	0.922^3^
WBC (/μl)^1^	541	5 (1-27)	80	6 (1-22)	240	6 (1-33)	157	6 (1-27)	19	3 (0-65)	45	2 (0-10)	**<0.001** ^3^
WBC ≥5/μl^2^	541	291 (54)	80	44 (55)	240	140 (58)	157	92 (59)	19	7 (37)	45	8 (18)	**<0.001** ^4^
WBC (/μl) in patients with CSF pleocytosis^1^	291	10 (5-40)	44	10 (6-28)	140	10 (5-43)	92	10 (5-32)	7	8 (5-65)	8	9 (5-27)	0.959^3^
Lymphocytes/plasma cells^2,7^	403	399 (99)	60	59 (98)	190	189 (100)	114	113 (99)	12	12 (100)	27	26 (96)	0.583^4^
Monocytes/macrophages^2,7^	403	165 (41)	60	21 (35)	190	84 (44)	114	46 (40)	12	4 (33)	27	9 (33)	0.616^4^
Neutrophils^2,7^	403	36 (9)	60	5 (8)	190	15 (8)	114	13 (11)	12	2 (17)	27	1 (4)	0.571^4^
Eosinophils^2,7^	403	0 (0)	60	0 (0)	190	0 (0)	114	0 (0)	12	0 (0)	27	0 (0)	NA
Basophils^2,7^	403	0 (0)	60	0 (0)	190	0 (0)	114	0 (0)	12	0 (0)	27	0 (0)	NA
No cells^2^	541	138	80	20 (25)	240	50 (21)	157	43 (27)	19	7 (37)	45	18 (40)	0.451^4^
Shadow cells^2^	541	287 (53)	80	45 (56)	240	135 (56)	157	82 (52)	19	8 (42)	45	17 (38)	0.166^4^
**Protein diagnostics**													
CSF TP (mg/l)^1^	540	390 (230-680)	80	355 (225-655)	239	380 (220-660)	157	410 (240-730)	19	450 (210-990)	45	400 (260-630)	**0.005** ^3^
CSF TP ≥ aURL^2,5^	539	103 (19)	80	12 (15)	238	40 (17)	157	36 (23)	19	8 (42)	45	7 (16)	**0.040** ^4^
CSF TP ≥ cURL^2,5^	540	185 (34)	80	21 (26)	239	76 (32)	157	61 (39)	19	10 (53)	45	17 (38)	0.112^4^
***Blood-CSF-barrier function***													
CSF albumin (mg/l)^1^	528	194 (109-418)	80	189 (104-321)	235	190 (108-419)	155	201 (113-448)	15	216 (130-437)	43	221 (114-422)	0.058^3^
Serum albumin (g/l)^1^	527	42.4 (33.8-50.3)	80	42.9 (32.0-49.3)	235	42.4 (34.5-50.6)	154	42.4 (34.0-50.1)	15	37.5 (24.4-47.5)	43	42.7 (34.5-51.2)	0.050^3^
Q_alb_^1^	527	4.7 (2.6-10.4)	80	4.5 (2.4-7.4)	235	4.6 (2.6-10.4)	154	4.9 (2.6-11.3)	15	5.6 (3.1-17.1)	43	4.9 (3.0-9.9)	**0.022** ^3^
Q_alb_ ≥ aURL^2,6^	527	37 (7)	80	2 (3)	235	14 (6)	154	15 (10)	15	4 (27)	43	2 (5)	**0.008** ^4^
Q_alb_ ≥ cURL^2,6^	527	138 (26)	80	11 (14)	235	63 (27)	154	48 (31)	15	6 (40)	43	10 (23)	**0.039** ^4^

Data are shown as ^1^median (5^th^-95^th^ percentile) or ^2^n (%). Group comparisons were performed by ^3^Kruskal-Wallis test or ^4^Pearson Chi-Quadrat test. ^5^For CSF TP, the cURL was <45 mg/l, the aURL was calculated according to the formula of McCudden et al. (Clin Chem 2017;63(12): 1856-1865). ^6^For Q_alb_, the cURL was calculated as “age/15+4” (Reiber et al. J Neurol Sci 2001;184:101–22), the aURL was determined as “age/25+8” (Hegen et al. Clin Chem Lab Med. 2016;54(2):285-92). ^7^Percentage of CSF samples with evidence of the respective leukocyte subtypes (lymphocytes/plasma cells, monocytes/macrophages, neutrophils, eosinophils and basophils) refers to CSF samples with any cells detectable by microscopy in the cytology (i.e. on the glass slide; n=403). P-values <0.05 are marked bold. Note that these are uncorrected P-values just for descriptive analysis.

aURL, age-dependent upper reference limit; CIS, clinically isolated syndrome; CSF, cerebrospinal fluid; cURL, conventional upper reference limit; MS, multiple sclerosis; NA, not appropriate; PPMS, primary progressive multiple sclerosis; Q_alb_, CSF/serum albumin quotient; RBC, red blood cell; RRMS, relapsing-remitting multiple sclerosis; SPMS, secondary progressive multiple sclerosis; TP, total protein; WBC, white blood cell.

**Figure 2:**
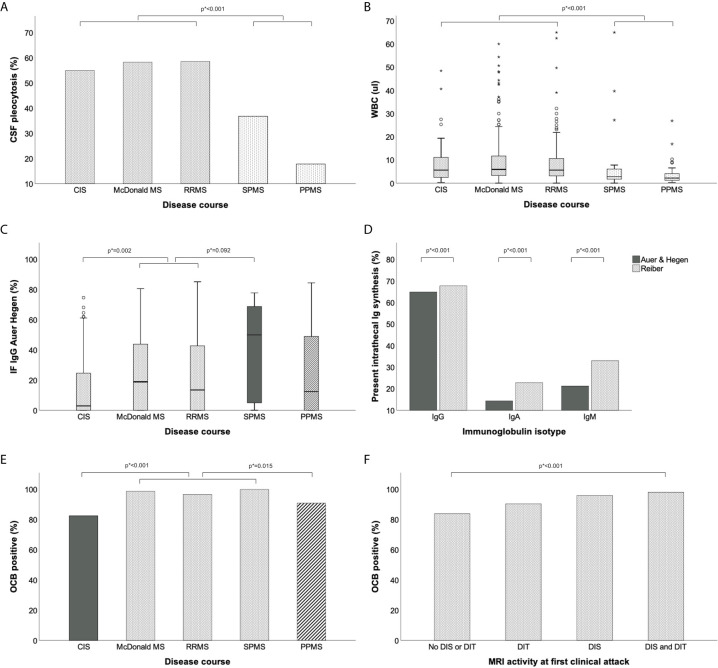
Routine CSF parameters in different MS disease courses. **(A)** Frequency of elevated WBC count (≥5/μl) is higher in relapsing than progressive MS. **(B)** Median WBC count is higher in relapsing than progressive MS. **(C)** Intrathecal IgG fraction is higher in secondary progressive than relapsing MS. **(D)** Frequency of intrathecal IgA and IgM synthesis is lower according to Auer & Hegen than Reiber formula. **(E, F)** Frequency of CSF-restricted OCB is highest in definite MS. Group comparisons were performed by Mann-Whitney-U test or Pearson Chi-squared test. P values (marked as p*) were corrected for multiple comparisons after Bonferroni. CIS, clinically isolated syndrome; CSF, cerebrospinal fluid; DIS, dissemination in space; dissemination in time; IF, intrathecal fraction; Ig, immunoglobulin; MRI, magnetic resonance imaging; MS, multiple sclerosis; OCB, oligoclonal bands; PPMS, primary progressive multiple sclerosis; RRMS, relapsing-remitting multiple sclerosis; SPMS, secondary progressive multiple sclerosis.

WBC count significantly differed between the various disease courses and showed a more than 2-fold increase in relapsing courses (CIS, McDonalds MS and RRMS) as compared to progressive courses (SPMS and PPMS) (**Figures 2A, B**). Also, WBC count negatively correlated with patients’ age (**Table S4**) and was statistically significantly higher in males (median 6/μl *versus* 5/μl, p=0.028). All these findings were confirmed in multivariate linear regression analysis adjusting for sex, age, disease duration and disease course (**Table S5**).

In patients with LP at first clinical attack, elevation of WBC count [odds ratio (OR) 2.9, 95% CI: 1.0 – 8.2, p=0.049] as well as the presence of monocytes/macrophages in CSF cytology (OR 7.3, 95% CI: 1.4 – 38.2, p=0.018) were predictive for the occurrence of a second clinical attack during follow-up (**Table 2**, **Table S10** and **Figure S1**).

**Table 2:** Cerebrospinal fluid findings in patients with a first clinical attack according to the occurrence of a second relapse during follow-up.

	No relapse		Relapse		P value
n	29		74		
**Cells and Cytology**					
RBC (/μl)^1^	29	0 (0-185)	74	0 (0-265)	0.806^3^
WBC (/μl)^1^	29	4 (1-27)	74	7 (2-25)	**0.009** ^3^
WBC ≥5/μl^2^	29	12 (41)	74	50 (68)	**0.015** ^4^
Lymphocytes/plasma cells^2,7^	21	21 (100)	64	63 (98)	0.564^4^
Monocytes/macrophages^2,7^	21	2 (10)	64	26 (41)	**0.009** ^4^
Neutrophils^2,7^	21	1 (5)	64	4 (6)	0.801^4^
Eosinophils^2,7^	21	0 (0)	64	0 (0)	NA
Basophils^2,7^	21	0 (0)	64	0 (0)	NA
No cells^2^	29	8 (28)	74	10 (14)	0.055^4^
Shadow cells^2^	29	13 (45)	74	40 (54)	0.399^4^
**Protein diagnostics**					
CSF TP (mg/l)^1^	29	350 (190-600)	74	360 (240-630)	0.448^3^
CSF TP ≥ aURL^2,5^	29	4 (14)	74	13 (18)	0.643^4^
CSF TP ≥ cURL^2,5^	29	7 (24)	74	23 (31)	0.485^4^
Q_alb_^1^	28	4.4 (2.3-9.1)	73	4.6 (2.4-8.8)	0.682^3^
Q_alb_ ≥ aURL^2,6^	28	1 (4)	73	2 (3)	0.826^4^
Q_alb_ ≥ cURL^2,6^	28	5 (18)	73	12 (16)	0.865^4^
***Intrathecal IgG synthesis***					
IF_Reiber_ >0^2^	28	15 (54)	73	49 (67)	0.206^4^
IF_Reiber_ (%)^1^	28	1 (0-67)	73	20 (0-68)	0.109^3^
IF_Auer&Hegen_ >0^2^	28	13 (46)	73	47 (64)	0.100^4^
IF_Auer&Hegen_ (%)^1^	28	0 (0-62)	73	15 (0-65)	0.101^3^
IgG index^1^	28	0.72 (0.44-1.88)	73	0.86 (0.5-2.05)	0.104^3^
IgG index >0.7^2^	28	15 (54)	73	49 (67)	0.206^4^
OCB positive^2^	29	24 (83)	74	69 (93)	0.106^4^
***Intrathecal IgA synthesis***					
IF_Reiber_ >0^2^	28	8 (29)	72	15 (21)	0.409^4^
IF_Reiber_ (%)^1^	28	0 (0-44)	72	0 (0-52)	0.836^3^
IF_Auer&Hegen_ >0^2^	28	4 (14)	72	9 (13)	0.812^4^
IF_Auer&Hegen_ (%)^1^	28	0 (0-33)	72	0 (0-42)	0.812^3^
***Intrathecal IgM synthesis***					
IF_Reiber_ >0^2^	28	9 (32)	73	20 (27)	0.637^4^
IF_Reiber_ (%)^1^	28	0 (0-71)	73	0 (0-57)	0.391^3^
IF_Auer&Hegen_ >0^2^	28	7 (25)	73	12 (16)	0.324^4^
IF_Auer&Hegen_ (%)^1^	28	0 (0-56)	73	0 (0-38)	0.395^3^

Data are shown as ^1^median (5^th^-95^th^ percentile) or ^2^n (%). Group comparisons were performed by ^3^Mann-Whitney U test or ^4^Pearson Chi-Quadrat test. ^5^For CSF TP, the cURL was <45 mg/l, the aURL was calculated according to the formula of McCudden et al. (Clin Chem 2017;63(12): 1856-1865). ^6^For Q_alb_, the cURL was calculated as “age/15+4” (Reiber et al. J Neurol Sci 2001;184:101–22), the aURL was determined as “age/25+8” (Hegen et al. Clin Chem Lab Med. 2016;54(2):285-92). ^7^Percentage of CSF samples with evidence of the respective leukocyte subtypes (lymphocytes/plasma cells, monocytes/macrophages, neutrophils, eosinophils and basophils) refers to CSF samples with any cells detectable by microscopy in the cytology (i.e. on the glass slide; n=85). P-values <0.05 are marked bold. Note that these are uncorrected P-values just for descriptive analysis.

aURL, age-dependent upper reference limit; cURL, conventional upper reference limit; CSF, cerebrospinal fluid; IF, intrathecal fraction; Ig, immunoglobulin; NA, not appropriate; OCB, oligoclonal bands; Q_alb_, CSF/serum albumin quotient, RBC, red blood cell, TP, total protein; WBC, white blood cell.

### Blood-CSF-Barrier Function

Q_alb_ was determined in 527 patients, had a median of 4.7 with an upper limit of 10.4 (95^th^ percentile) and was increased in 37 (7%) patients using the aURL as opposed to 138 (26%) using the cURL. CSF total protein was determined in 540 patients, showed a median of 390 mg/L (95^th^ percentile: 680 mg/L) and was elevated in 103 (19%) patients using the aURL compared to 185 (34%) patients applying the cURL (**Table 1A**). Both, Q_alb_ and CSF-TP correlated with patients’ age (**Table S4**) and were statistically significantly higher in males (median 440 mg/L *versus* 370 mg/L, p<0.001). In multivariate linear regression analysis, the effect of sex and age was confirmed, while disease course had no impact on Q_alb_ levels (**Table S6**, **S7**).

### Intrathecal Ig Synthesis

An intrathecal IgG synthesis was found in 342 (65%) of 527 patients using the Auer & Hegen formula and in 357 (68%) applying the Reiber formula. The IgG index was elevated (>0.7) in 362 (69%) of patients (**Table 1B**). While the frequencies of intrathecal IgG synthesis were similar using these different quantitative methods, the intrathecal IgA and IgM synthesis were less frequently observed according to the Auer & Hegen (14% and 24%) than the Reiber formula (23% and 33%; p<0.001; **Figure 2D**, **Table 1B**). OCB as qualitative method to detect intrathecal IgG synthesis were positive in 503 (95%) of 529 patients (**Table 1B**). The frequencies of intrathecal IgG, IgA and IgM synthesis determined by the different quantitative formulae according to OCB status, i.e. within the OCB positive and OCB negative patients, are provided in **Table S11**.

**Table 1B:** Cerebrospinal fluid findings in multiple sclerosis and according to different disease courses: Intrathecal Ig synthesis.

	Whole cohort		CIS		McDonalds MS		RRMS		SPMS		PPMS		P value
	n		n		n		n		n		n		
***Immunoglobulins***													
CSF IgG (mg/l)^1^	528	46.1 (19.3-138.0)	80	32.9 (15.2-101.6)	235	46.3 (20.2-127.0)	155	50.9 (18.8-141.0)	15	75.2 (26.8-342.0)	43	49.2 (20.1-152.0)	**<0.001** ^3^
CSF IgA (mg/l)^1^	518	3.20 (0.20-8.90)	79	2.90 (0.20-6.90)	231	3.10 (0.20-8.80)	152	3.60 (0.20-9.40)	15	4.20 (1.80-16.10)	41	3.20 (0.20-9.40)	**0.004** ^3^
CSF IgM (mg/l)^1^	528	0.90 (0.15-4.50)	80	0.70 (0.15-2.25)	235	0.90 (0.15-4.70)	155	1.00 (0.15-4.70)	15	0.80 (0.15-9.90)	43	0.80 (0.15-4.00)	**0.032** ^3^
Serum IgG (g/l)^1^	527	10.30 (7.01-14.10)	80	10.10 (6.90-14.20)	235	10.40 (7.06-14.30)	154	10.30 (7.08-14.20)	15	9.53 (5.34-12.90)	43	10.2 (8.42-13.60)	0.449^3^
Serum IgA (g/l)^1^	525	1.84 (0.83-3.52)	80	1.68 (0.92-3.51)	234	1.83 (0.83-3.46)	154	1.92 (0.68-3.93)	15	2.28 (0.67-3.08)	42	1.87 (1.03-3.59)	0.166^3^
Serum IgM (g/l)^1^	527	1.21 (0.53-2.71)	80	1.17 (0.50-3.09)	235	1.26 (0.55-2.43)	154	1.19 (0.52-2.86)	15	1.11 (0.60-2.09)	43	1.39 (0.48-3.27)	0.786^3^
***Intrathecal IgG synthesis***													
IF_Reiber_ >0^2^	527	357 (68)	80	44 (55)	235	168 (72)	154	107 (70)	15	11 (73)	43	27 (63)	0.079^4^
IF_Reiber_ (%)^1^	527	20 (0-71)	80	13 (0-66)	235	22 (0-69)	154	19 (0-71)	15	51 (0-78)	43	15 (0-72)	**0.019** ^3^
IF_Auer&Hegen_ >0^2^	527	342 (65)	80	43 (54)	235	157 (67)	154	105 (68)	15	11 (73)	43	26 (61)	0.177^4^
IF_Auer&Hegen_ (%)^1^	527	15 (0-69)	80	3 (0-62)	235	19 (0-67)	154	13 (0-68)	15	50 (0-78)	43	12 (0-70)	**0.004** ^3^
IgG index^1^	527	0.86 (0.50-2.35)	80	0.75 (0.47-1.90)	235	0.89 (0.52-2.22)	154	0.86 (0.51-2.29)	15	1.44 (0.52-3.37)	43	0.85 (0.48-2.42)	**0.002** ^3^
IgG index >0.7^2^	527	362 (69)	80	45 (56)	235	165 (70)	154	115 (75)	15	11 (73)	43	26 (60)	**0.039** ^4^
OCB positive^2^	529	503 (95)	80	66 (83)	240	237 (99)	153	148 (97)	12	12 (100)	44	40 (91)	**<0.001** ^4^
***Intrathecal IgA synthesis***													
IF_Reiber_ >0^2^	517	118 (23)	79	17 (22)	231	53 (23)	151	30 (20)	15	7 (47)	41	11 (27)	0.196^4^
IF_Reiber_ (%)^1^	517	0 (0-50)	79	0 (0-52)	231	0 (0-50)	151	0 (0-53)	15	0 (0-26)	41	0 (0-41)	0.479^3^
IF_Auer&Hegen _>0^2^	517	74 (14)	79	8 (10)	231	36 (16)	151	22 (15)	15	3 (20)	41	5 (12)	0.738^4^
IF_Auer&Hegen_ (%)^1^	517	0 (0-38)	79	0 (0-42)	231	0 (0-37)	151	0 (0-41)	15	0 (0-6)	41	0 (0-31)	0.375^3^
***Intrathecal IgM synthesis***													
IF_Reiber_ >0^2^	527	174 (33)	80	22 (28)	235	85 (36)	154	50 (33)	15	4 (27)	43	13 (30)	0.626^4^
IF_Reiber_ (%)^1^	527	0 (0-76)	80	0 (0-73)	235	0 (0-76)	154	0 (0-78)	15	0 (0-78)	43	0 (0-64)	0.525^3^
IF_Auer&Hegen _>0^2^	527	112 (21)	80	14 (18)	235	53 (23)	154	34 (22)	15	2 (13)	43	9 (21)	0.820^4^
IF_Auer&Hegen_ (%)^1^	527	0 (0-62)	80	0 (0-58)	235	0 (0-62)	154	0 (0-67)	15	0 (0-66)	43	0 (0-44)	0.565^3^

Data are shown as ^1^median (5^th^-95^th^ percentile) or ^2^n (%). Group comparisons were performed by ^3^Kruskal-Wallis test or ^4^Pearson Chi-Quadrat test. P-values < 0.05 are marked bold. Note that these are uncorrected P-values just for descriptive analysis.

CIS, clinically isolated syndrome; CSF, cerebrospinal fluid; IF, intrathecal fraction; Ig, immunoglobulin; MS, multiple sclerosis; OCB, oligoclonal bands; PPMS, primary progressive multiple sclerosis; RRMS, relapsing-remitting multiple sclerosis; SPMS, secondary progressive multiple sclerosis.

Intrathecal fraction of IgG significantly differed among the various disease courses with the lowest values in patients with CIS and the highest in SPMS (**Figure 2C**); this was also confirmed in multivariate regression analysis (**Table S8**). Intrathecal fraction of IgA and IgM were similar across disease courses (**Table 1B**). The prevalence of OCB was higher in patients with definite diagnosis of MS (**Figure 2E**). In patients with LP at first clinical attack, prevalence of OCB was associated with diagnostic brain MRI criteria and highest in patients fulfilling both DIT and DIS (**Figure 2F**, **Tables 3** and **S9**).

**Table 3:** Cerebrospinal fluid findings in patients with a first clinical attack according to MRI criteria of DIS and DIT.

	Only DIS		Only DIT		DIS & DIT		No DIS and DIT		P value
**Cells and Cytology**									
RBC (/μl)^1^	75	0 (0-41)	21	0 (0-83)	168	0 (0-85)	56	0 (0-265)	0.934^3^
WBC (/μl)^1^	75	4 (1-25)	21	6 (2-41)	168	7 (1-37)	56	6 (1-19)	**0.023** ^3^
WBC ≥5/μl^2^	75	34 (45)	21	11 (52)	168	106 (63)	56	33 (59)	0.073^4^
Lymphocytes/plasma cells^2,7^	57	56 (98)	17	17 (100)	134	134 (100)	42	41 (98)	0.363^4^
Monocytes/macrophages^2,7^	57	21 (37)	17	4 (24)	134	63 (47)	42	17 (40)	0.219^4^
Neutrophils^2,7^	57	4 (7)	17	1 (6)	134	11 (8)	42	4 (10)	0.956^4^
Eosinophils^2,7^	57	0 (0)	17	0 (0)	134	0 (0)	42	0 (0)	NA
Basophils^2,7^	57	0 (0)	17	0 (0)	134	0 (0)	42	0 (0)	NA
No cells^2^	75	18 (24)	21	4 (19)	168	34 (20)	56	14 (25)	0.420^4^
Shadow cells^2^	75	38 (51)	21	12 (57)	168	98 (58)	56	32 (57)	0.736^4^
**Protein diagnostics**									
CSF TP (mg/l)^1^	75	380 (200-660)	21	330 (220-550)	167	380 (230-660)	56	355 (230-640)	0.367^3^
CSF TP ≥ aURL^2,5^	75	14 (19)	21	4 (19)	166	27 (16)	56	7 (13)	0.798^4^
CSF TP ≥ cURL^2,5^	75	26 (35)	21	5 (24)	167	51 (31)	56	15 (27)	0.700^4^
Q_alb_^1^	73	4.6 (2.5-9.7)	21	4.6 (2.4-6.5)	165	4.6 (2.7-10.8)	56	4.3 (2.5-7.1)	0.257^3^
Q_alb_ ≥ aURL^2,6^	73	4 (6)	21	0 (0)	165	11 (7)	55	1 (2)	0.352^4^
Q_alb_ ≥ cURL^2,6^	73	16 (22)	21	2 (10)	165	49 (30)	56	7 (13)	**0.021** ^4^
***Intrathecal IgG synthesis***									
IF_Reiber_ >0^2^	73	52 (71)	21	12 (57)	165	116 (70)	56	32 (57)	0.187^4^
IF_Reiber_ (%)^1^	73	22 (0-66)	21	23 (0-64)	165	21 (0-69)	56	15 (0-67)	0.149^3^
IF_Auer&Hegen_ >0^2^	73	48 (66)	21	12 (57)	165	109 (66)	56	31 (55)	0.458^4^
IF_Auer&Hegen_ (%)^1^	73	18 (0-64)	21	14 (0-62)	165	19 (0-67)	56	5 (0-62)	0.066^3^
IgG index^1^	73	0.89 (0.52-2.02)	21	0.84 (0.48-1.91)	165	0.89 (0.51-2.22)	56	0.76 (0.46-1.89)	**0.046** ^3^
IgG index >0.7^2^	73	50 (69)	21	12 (57)	165	115 (70)	56	33 (59)	0.368^4^
OCB positive^2^	75	72 (96)	21	19 (91)	168	165 (98)	56	47 (84)	**<0.001** ^4^
***Intrathecal IgA synthesis***									
IF_Reiber_ >0^2^	72	18 (25)	21	6 (29)	162	36 (22)	55	10 (18)	0.731^4^
IF_Reiber_ (%)^1^	72	0 (0-58)	21	0 (0-44)	162	0 (0-43)	55	0 (0-52)	0.896^3^
IF_Auer&Hegen_ >0^2^	72	13 (18)	21	4 (19)	162	24 (15)	55	3 (6)	0.188^4^
IF_Auer&Hegen_ (%)^1^	72	0 (0-53)	21	0 (0-33)	162	0 (0-31)	55	0 (0-42)	0.742^3^
***Intrathecal IgM synthesis***									
IF_Reiber_ >0^2^	73	24 (33)	21	6 (29)	165	62 (38)	56	15 (27)	0.465^4^
IF_Reiber_ (%)^1^	73	0 (0-79)	21	0 (0-71)	165	0 (0-71)	56	0 (0-74)	0.631^3^
IF_Auer&Hegen_ >0^2^	73	18 (25)	21	4 (19)	165	36 (22)	56	9 (16)	0.683^4^
IF_Auer&Hegen_ (%)^1^	73	0 (0-68)	21	0 (0-56)	165	0 (0-58)	56	0 (0-59)	0.579^3^

Data are shown as ^1^median (5^th^-95^th^ percentile) or ^2^n (%). Group comparisons were performed by ^3^Kruskal-Wallis test or ^4^Pearson Chi-Quadrat test. ^5^For CSF TP, the cURL was <45 mg/l, the aURL was calculated according to the formula of McCudden et al. (Clin Chem 2017;63(12): 1856-1865). ^6^For Q_alb_, the cURL was calculated as “age/15+4” (Reiber et al. J Neurol Sci 2001;184:101–22), the aURL was determined as “age/25+8” (Hegen et al. Clin Chem Lab Med. 2016;54(2):285-92). ^7^Percentage of CSF samples with evidence of the respective leukocyte subtypes (lymphocytes/plasma cells, monocytes/macrophages, neutrophils, eosinophils and basophils) refers to CSF samples with any cells detectable by microscopy in the cytology (i.e. on the glass slide; n=250). P-values <0.05 are marked bold. Note that these are uncorrected P-values just for descriptive analysis.

aURL, age-dependent upper reference limit; cURL, conventional upper reference limit; CSF, cerebrospinal fluid; DIS, dissemination in space; DIT, dissemination in time; IF, intrathecal fraction; Ig, immunoglobulin; NA, not appropriate; OCB, oligoclonal bands; Q_alb_, CSF/serum albumin quotient, RBC, red blood cell, TP, total protein; WBC, white blood cell.

### The Added Diagnostic Value of OCB

Of 320 patients with LP at first clinical attack, 75 (23%) patients showed DIS on brain MRI. Of those, 72 (96%) had CSF-restricted OCB and, thus, finally fulfilled the diagnostic criteria for MS. In 43 patients with PPMS and available MRI data, 29 (67%) patients fulfilled only the criteria for DIS. Of those, 26 (90%) had CSF-restricted OCB, and, thus, were diagnosed as PPMS.

## Discussion

In this study, we showed that CSF profiles differ between the various MS disease courses and that certain CSF abnormalities occur less frequently than previously reported. This might be due to several reasons, such as the revisions of MS diagnostic criteria as well as the novel CSF RL.

We observed blood-CSF-barrier disturbance as indicated by elevation of the Q_alb_ in 7% of the whole population applying aURL as opposed to 26% using cURL. In multivariate analysis, we showed that absolute changes in Q_alb_ are associated with patients’ age and sex, and not with the disease course (**Table S6**). It has been long known that Q_alb_ increases with age (6) and that CSF protein levels are slightly higher in males (22). Previously reported frequencies of elevated Q_alb_ in up to 15% of MS patients (24) are most likely due to a significant amount of false-positive results obtained by the cURL. Indeed, elevated Q_alb_ levels using the cURL have been reported in 15% of patients without evidence of any neurological disorder (ruled out by clinical, laboratory and imaging diagnostics) (25). As Q_alb_ is elevated in almost every other patient with myelin oligodendrocyte glycoprotein (MOG)-associated disorders (MOGAD) (26) or aquaporin-4 positive Neuromyelitis optica spectrum disorder (NMOSD) (27), a correct age-dependent interpretation is important in differential diagnosis of CNS demyelinating diseases.

For CSF-TP, the outdated cURL of 450 mg/L is still widely adopted in over 85% of clinical centers worldwide (28) and quoted in clinical practice guidelines (6). As CSF-TP level also increases with age, two recent studies on over 6000 CSF samples published aURL (21, 22) in order to overcome a high rate of false-positive results. We observed that using the aURL reduced the frequency of elevated CSF-TP from 34% to 19% in the whole cohort. Furthermore, Q_alb_ and CSF-TP are both elevated in case of blood-CSF barrier dysfunction (6). Whereas Q_alb_ is the gold standard to reflect all the effects on the passage of proteins from blood into the CSF, including diffusivity (predominantly across blood-CSF barrier) and CSF flow (7, 29, 30), CSF-TP is also influenced by an intrathecal protein synthesis (31). A discrepancy between elevated CSF-TP and normal Q_alb_ was observed in 12% of patients and might be explained at least to some extent by different concentration of intrathecal Ig synthesis. Indeed, the intrathecal fraction of IgG determined by the Auer & Hegen formula as well as the IgG index were significantly higher in patients with elevated CSF-TP but normal Q_alb_ as compared to patients with both normal CSF-TP and normal Q_alb_ (IgG index 0.97 *vs*. 0.84; p=0.003 and IF IgG 23% *vs*. 13%, p=0.005). With regard to the URL, previous studies have already shown that agreement between Q_alb_ and CSF-TP is higher when aURL were used (21).

The proof of an intrathecal IgG synthesis is one of the hallmarks of MS. The gold standard is isoelectric focusing and subsequent immunoblotting to detect OCB allowing qualitative IgG detection (i.e. positive *versus* negative result) (3). In line with previous studies, we observed a sensitivity of >95% in definite MS (3, 19) and 83% in patients with CIS (32).

In patients with a first demyelinating event, we could show that OCB were associated with the extent of brain MRI activity. While OCB were demonstrated in only 84% of patients not fulfilling both DIS and DIT by means of MRI, the highest frequency of positive OCB was found in patients with DIS and DIT (98%). This is of special interest, as OCB status does not add to diagnostic certainty in the second scenario because almost all patients who fulfilled MRI criteria were OCB positive. On the other hand, OCB was a significant contributor to diagnosis in patients not meeting MRI criteria. Of 75 patients with a first clinical event who showed only DIS on brain MRI, 72 (96%) had CSF-restricted OCB and, thus, fulfilled the diagnostic criteria for MS. Importantly, it has to be stated that patients with a first demyelinating event without DIS and DIT might not develop MS in future or even might suffer from a disorder other than MS.

Similarly, of 29 patients with PPMS fulfilling DIS only, 26 (90%) had CSF-restricted OCB, and, thus, were diagnosed as PPMS. This substantiates CSF-restricted OCB as a cumulative marker for DIT being constantly detectable during disease course – as opposed to contrast-enhancing lesions which are only temporarily detectable on MRI. This concept has already been incorporated into the 2017 McDonald Criteria (1).

Another approach for detecting intrathecal Ig production is the quantitative determination of Ig concentrations in the CSF and serum followed by calculating the intrathecal Ig fraction by means of various formulae (6). This allows the determination of intrathecal IgA, IgM and IgG production. A widely used formula was developed by Reiber et al. (23, 33). However, the hyperbolic formulae of Reiber do return false-positive results, particularly for IgA and IgM. This is evidenced by studies showing intrathecal Ig synthesis in conditions usually not associated with a local humoral immune response (34), as well as intrathecal IgG synthesis in OCB negative patients (35). The German Society for Cerebrospinal Fluid Diagnostics and Neurochemistry recommended considering intrathecal IgA or IgM synthesis of less than 10% as non-pathological (36). That is why a new formula has been developed to reduce false positive results (12). Here, we show that the quantitative intrathecal IgG synthesis is similar using the Reiber and Auer & Hegen formula, however, the former returns more frequently positive results for IgM and IgA. Interestingly, the amount of intrathecal IgG synthesis differed between disease courses with highest rates in SPMS. This confirms previous small studies that showed higher intrathecal IgG concentrations in SPMS *versus* relapsing MS (37). As we observed a significant impact of disease course on intrathecal IgG production even after adjusting for disease duration in the multivariate model, one might hypothesize that the extent of (previously occurred) inflammation in patients with SPMS drives the higher IgG synthesis. Earlier speculated association with pathophysiology of progression remains elusive, as we did not have the Expanded Disability Status Scale Score of all patients at the time of LP available to perform this analysis. With regard to intrathecal IgA synthesis, our findings show that it is uncommon in CIS and MS patients and should prompt to consider differential diagnoses. In contrast, intrathecal IgM synthesis is still present in a significant proportion of patients (>20%). To know about the correct frequency of patients with intrathecal IgM synthesis is of interest, as its presence has been associated with a more aggressive disease course (38). At this point, we want to state that delineating intrathecal IgM and IgA synthesis remains difficult, as for the establishment of any quantitative formula a reliable endpoint (e.g. OCB in terms of IgG) does not exist for IgM and IgA. While previously performed studies on selected “test cohorts” served as basis for calculating the Reiber and Auer & Hegen formulae, respectively, and higher specificity was shown by the Auer & Hegen formula, it is evident to conclude that the lower sensitivity of the Auer & Hegen formula is due to its higher specificity. However, further studies that compare the performance of both formulae with regard to the presence of oligoclonal IgM bands would be desirable.

With regard to WBC count, we also confirmed previous studies that found roughly half of MS patients displaying CSF pleocytosis (up to 40/μl) (4, 7, 39). Furthermore, we found that WBC counts are generally higher in relapsing than in progressive disease courses. Although the differential cell count is dominated by lymphocytes in MS, we showed that in some cases neutrophils may occur. Knowledge on leukocyte subtypes is of importance for differential diagnosis of MS, as NMOSD or MOGAD are occasionally associated with a predominant presence of neutrophils (27, 40). Interestingly, we observed that the presence of monocytes/macrophages was associated with conversion to CDMS. This underlines findings on Chitinase 3-like 1 (CHI3L1), a glycoprotein secreted by macrophages (41), which was determined as a biomarker in MS (42–44) and which was also associated with the conversion from CIS to CDMS (45).

There are some limitations of the present study that arise mainly from its retrospective design. E.g., CSF diagnostics and MRI were performed as part of routine diagnostics, therefore, bias due to different image acquisition (e.g. field strength) or variable duration between MRI and LP cannot be excluded. At this point, we also want to state that the presence of certain leukocyte subtypes was retrieved from routine CSF reports. It cannot be excluded that the frequency of cell types that are infrequently found in the CSF of MS patients are underestimated. Due to this shortcoming, we grouped plasma cells in the same category as lymphocytes and macrophages in the same as monocytes. That is why an analysis of different states of cell activation was not feasible, e.g. to separate lymphocytes from activated lymphocytes and plasma cells. Whereas the differentiation of leukocyte subtypes is usually simple and the simple evaluation of their presence in a cytological preparation reliable; this is not the case for shadow cells. Shadow cells are supposed to reflect apoptotic cells and even though we referred to a common definition (17), their determination remains rater-dependent. Also, it cannot be excluded that cytospin preparation impacts on formation of shadow cells. Further studies are required that investigate the association between the presence of shadow cells as determined by microscopy and other markers of apoptosis in order to illuminate their role in MS.

In the present work, we confirmed previous studies on the CSF profile in MS, including e.g. the upper limit of WBC count (up to 40/μl) or the high sensitivity of OCB (reaching 95%) (4, 6). We also showed that CSF profile in MS varies across disease courses and that some parameters are associated with early MS disease activity. For the first time, we emphasize that certain abnormalities, e.g. blood-CSF-barrier dysfunction and intrathecal IgA/IgM synthesis are less frequent in MS when applying novel state-of-the-art RL. These findings are of high clinical relevance, as a correct interpretation of CSF results has an essential impact on the differential diagnosis of CNS demyelinating diseases.

## Data Availability Statement

The raw data supporting the conclusions of this article will be made available by the authors, without undue reservation.

## Ethics Statement

Ethical review and approval were not required for the study on human participants in accordance with the local legislation and institutional requirements. Written informed consent for participation was not required for this study in accordance with the national legislation and the institutional requirements.

## Author Contributions

KB has participated in acquisition and interpretation of data, and in drafting the manuscript. GB has participated in acquisition of data and in reviewing the manuscript for intellectual content. MA has participated in reviewing the manuscript for intellectual content. FP has participated in reviewing the manuscript for intellectual content. AZ has participated in reviewing the manuscript for intellectual content. TB has participated in reviewing the manuscript for intellectual content. FD has participated in reviewing the manuscript for intellectual content. HH has participated in the conception and design of the study, acquisition, statistical analysis and interpretation of the data, and in drafting the manuscript. All authors contributed to the article and approved the submitted version.

## Conflict of Interest

KB has participated in meetings sponsored by and received travel funding from Roche and Biogen. GB has participated in meetings sponsored by, received speaker honoraria or travel funding from Biogen, Celgene, Lilly, Merck, Novartis, Sanofi-Genzyme and Teva, and received honoraria for consulting Biogen, Celgene, Merck, Novartis, Roche and Teva. MA received speaker honoraria and/or travel grants from Biogen, Merck, Novartis and Sanofi. FP has participated in meetings sponsored by, received honoraria (lectures, advisory boards, consultations) or travel funding from Bayer, Biogen, Merck, Novartis, Sanofi-Genzyme, Teva, Celgene and Roche. Her institution has received research grants from Roche. AZ has participated in meetings sponsored by, received speaking honoraria or travel funding from Biogen, Merck, Sanofi-Genzyme and Teva. TB has participated in meetings sponsored by and received honoraria (lectures, advisory boards, consultations) from pharmaceutical companies marketing treatments for MS: Allergan, Biogen, Biologix, Bionorica, Celgene, Eisei, MedDay, Merck, Novartis, Roche, Sanofi-Genzyme, Teva, UCB. His institution has received financial support in the past 12 months by unrestricted research grants (Bayer, Biogen, Merck, Novartis, Roche, Sanofi-Genzyme, Teva) and for participation in clinical trials in multiple sclerosis sponsored by Alexion, Bayer, Biogen, Merck, Novartis, Roche, Sanofi-Aventis, Teva. FD has participated in meetings sponsored by or received honoraria for acting as an advisor/speaker for Almirall, Alexion, Biogen, Celgene, Genzyme-Sanofi, Merck, Novartis Pharma, Roche, and TEVA ratiopharm. His institution has received research grants from Biogen and Genzyme Sanofi. He is section editor of the MSARD Journal (Multiple Sclerosis and Related Disorders). HH has participated in meetings sponsored by, received speaker honoraria or travel funding from Bayer, Biogen, Merck, Novartis, Sanofi-Genzyme, Siemens, Teva, and received honoraria for acting as consultant for Teva and Biogen.
