# Supplementary material for: Cerebrospinal Fluid Findings in 541 Patients With Clinically Isolated Syndrome and Multiple Sclerosis: A Monocentric Study
Source: Front Immunol. 2021 Jun 17;12:675307. doi: 10.3389/fimmu.2021.675307 (PMC8248497; doi:10.3389/fimmu.2021.675307)
Supplement: Supplementary file 1 [file DataSheet_1.pdf]

Figure S1: Time to relapse depending on CSF cells

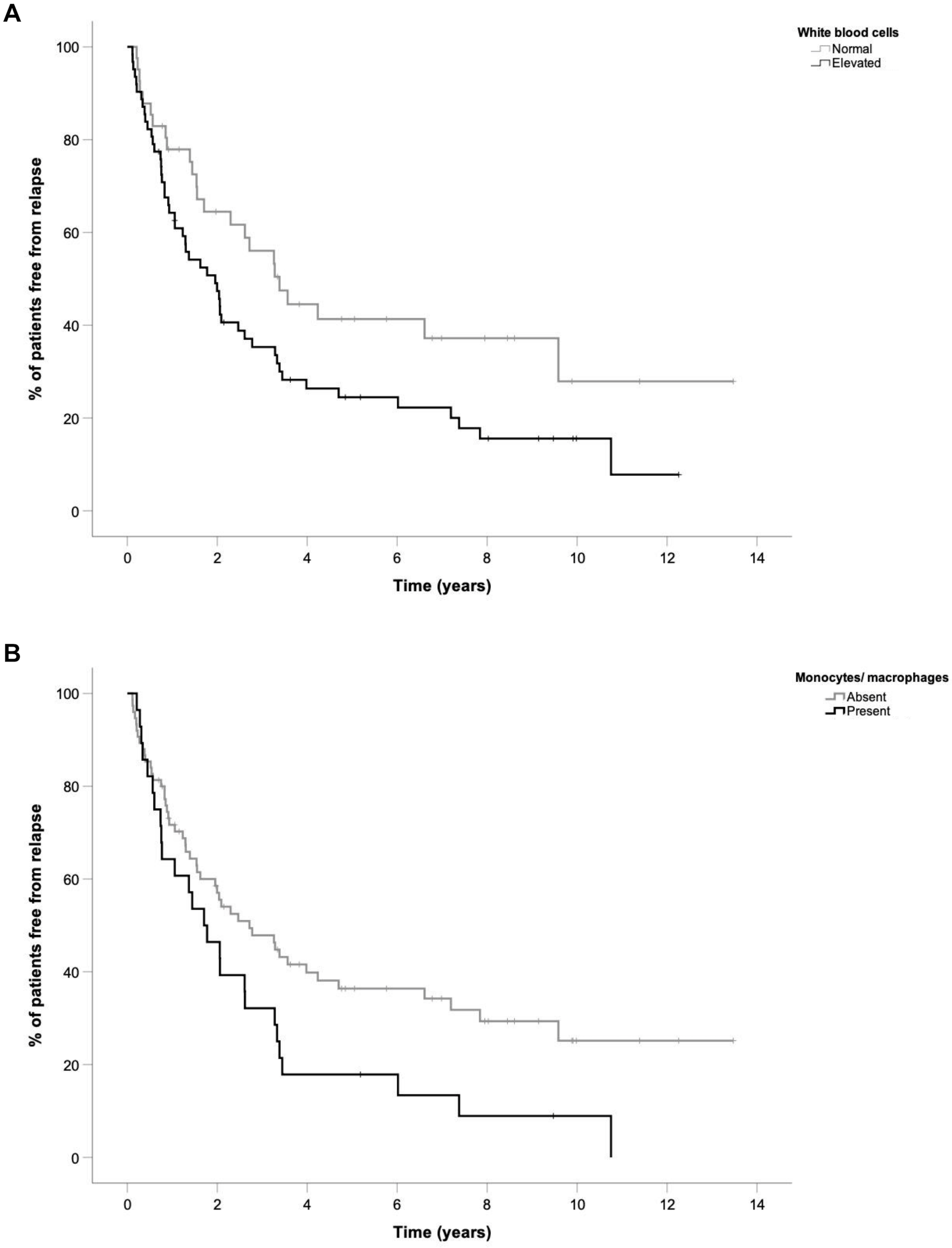

Kaplan-Meier survival curves for the time to relapse depending on

(A) CSF pleocytosis (Log Rank test: p=0.032) and
(B) presence of monocytes/ macrophages in CSF cytology (Log Rank test: p=0.027).

CSF, cerebrospinal fluid
