# Supplementary material for: Cerebrospinal Fluid Findings in 541 Patients With Clinically Isolated Syndrome and Multiple Sclerosis: A Monocentric Study
Source: Front Immunol. 2021 Jun 17;12:675307. doi: 10.3389/fimmu.2021.675307 (PMC8248497; doi:10.3389/fimmu.2021.675307)
Supplement: Supplementary file 2 [file DataSheet_2.pdf]

Supplementary methods

Auer & Hegen formulae

$$Q_{lim}(IgX) = a \times Qalb^{b} \quad (1)$$

Ig isotypes	a	b
IgG	0.882	1.035
IgA	0.819	1.076
IgM	1.845	1.340

$$IgX_{int} = (Q_{IgX} - Q_{lim\ IgX}) \times IgX_{ser} \quad (2)$$

$$Ig_{IF} = \frac{IgX_{int}}{IgX_{CSF}} \times 100 \quad (3)$$

Formula (1): The Q_{alb}-based cut-off Ig quotient ($Q_{lim\ IgX}$) is calculated by inserting the empirically determined values for *a* and *b*.

Formula (2): The amount of intrathecally synthesised Ig (IgX_{int}) is calculated.

Formula (3): The percentage of an intrathecally synthesised Ig fraction (Ig_{IF}) in the CSF is calculated.

Reiber formulae

$$Q_{lim}(IgX) = \frac{a}{b} \times \sqrt{{Q_{alb}}^{2} + b^{2}} - c \quad (1)$$

Ig isotypes	a/b	$b^2\times10^6$	$c\times10^3$
IgG	0.93	6	1.7
IgA	0.77	23	3.1
IgM	0.67	120	7.1

$$IgX_{int} = (Q_{IgX} - Q_{lim\ IgX}) \times IgX_{ser} \quad (2)$$

$$Ig_{IF} = \frac{IgX_{int}}{IgX_{CSF}} \times 100 \quad (3)$$

Formula (1): The Q_{alb}-based cut-off Ig quotient ($Q_{lim\ IgX}$) is calculated by inserting the empirically determined values for *a*, *b* and *c*. For convenience, the values for a/b, $b^2\times10^6$ and $c\times10^3$ are provided.

Formula (2): The amount of intrathecally synthesised Ig (IgX_{int}) is calculated.

Formula (3): The percentage of an intrathecally synthesised Ig fraction (Ig_{IF}) in the CSF is calculated.

Abbreviations: Ig, immunoglobulin; IgX_{CSF}, Ig concentration in CSF; IgX_{ser}, Ig concentration in serum; Q_{alb}, CSF/serum albumin quotient; Q_{IgX}, CSF/serum Ig quotient
