# Supplementary material for: Cerebrospinal Fluid Findings in 541 Patients With Clinically Isolated Syndrome and Multiple Sclerosis: A Monocentric Study
Source: Front Immunol. 2021 Jun 17;12:675307. doi: 10.3389/fimmu.2021.675307 (PMC8248497; doi:10.3389/fimmu.2021.675307)
Supplement: Supplementary file 3 [file DataSheet_3.pdf]

Table S1: Demographic and clinical characteristics of multiple sclerosis patients according to different disease courses

	Whole cohort		CIS		McDonalds MS		RRMS		SPMS		PPMS		P value
	n		n		n		n		n		n		
**Demographics**													
Age at disease onset (years)[1]	540	31 (19-48)	80	29 (21-52)	239	32 (20-47)	157	28 (18-44)	19	31 (15-48)	45	43 (28-58)	**<0.001**[3]
Age at LP (years)[1]	541	34 (21-54)	80	29 (21-52)	240	33 (21-48)	157	34 (21-51)	19	47 (32-69)	45	47 (32-63)	**<0.001**[3]
Sex (females)[2]	541	376 (70)	80	49 (61)	240	176 (73)	157	115 (73)	19	13 (68)	45	23 (51)	**0.014**[4]
**Clinical characteristics**													
Disease duration until LP (years) [1]	540	0.27 (0.01-16.2)	80	0.03 (0-0.5)	239	0.05 (0.01-1.5)	157	3.7 (0.3-20.0)	19	17.2 (0.8-48.2)	45	2.9 (0.4-15.2)	**<0.001**[3]
**MRI findings at baseline**													
DIS/ no DIT by MRI[2]			80	3 (4)	240	72 (30)							
DIT/ no DIS by MRI[2]			80	21 (26)	0	0 (0)							
DIS & DIT by MRI[2]			80	0 (0)	240	168 (70)							
DIS by MRI and DIT by OCB[2]			80	0 (0)	240	72 (30)							

Legend:

Data are shown as [1]median (5th-95th percentile) or [2]n (%). Group comparison were performed by [3]Kruskal-Wallis test or [4]Pearson Chi-Quadrat test. P-values <0.05 are marked bold.

Abbreviations: CIS, clinically isolated syndrome; DIS, dissemination in space; DIT, dissemination in time; LP, lumbar puncture; MRI, magnetic resonance imaging; MS, multiple sclerosis; OCB, oligoclonal bands; PPMS, primary progressive multiple sclerosis; RRMS, relapsing-remitting multiple sclerosis; SPMS, secondary progressive multiple sclerosis

Table S2: Demographic and clinical characteristics of patients with a first clinical attack according to MRI criteria of DIS and DIT

	DIS		DIT		DIS & DIT		No DIS or DIT		P value
	n		n		n		n		
**Demographics**									
Age at disease onset (years)[1]	75	35 (21-50)	21	32 (19-57)	167	30 (20-47)	56	28 (21-49)	**<0.001**[3]
Age at LP (years)[1]	75	36 (21-51)	21	33 (19-57)	168	30 (21-47)	56	28 (21-49)	**<0.001**[3]
Sex (female)[2]	75	60 (80)	21	12 (57)	168	117 (70)	56	36 (64)	0.106[4]
**Clinical characteristics**									
Disease duration until LP (months)	75	0.9 (0.1-37.4)	21	0.3 (0-6.2)	167	0.6 (0.1-16.9)	56	0.6 (0-5.5)	0.848
2nd relapse[2]	51	33 (65)	15	12 (80)	145	117 (81)	37	29 (78)	0.134[4]
Disease duration until 2nd relapse (years)[1]	33	1.7 (0.2-9.8)	12	1.5 (0.2-7.2)	116	1.3 (0.2-8.1)	29	1.8 (0.2-6.6)	0.933[3]

Legend:

Data are shown as [1]median (5th-95th percentile) or [2]n (%). Group comparison were performed by [3]Kruskal-Wallis test or [4]Pearson Chi-Quadrat test. P-values <0.05 are marked bold.

Abbreviations: DIS, dissemination in space; DIT, dissemination in time; LP, lumbar puncture

Table S3: Demographic and clinical characteristics of patients with a first clinical attack according to the occurrence of a second relapse during follow-up

	No relapse	Relapse	P value
n	29	74	
**Demographics**			
Age at disease onset (years)[1]	34 (20-42)	31 (19-46)	0.514[3]
Age at LP (years)[1]	34 (20-42)	31 (19-46)	0.562[3]
Sex (female)[2]	21 (72)	53 (72)	0.936[4]
**MRI findings at baseline**			
DIS[2]	18 (62)	33 (45)	0.278[4]
DIT[2]	3 (10)	12 (16)	
No DIS no DIT[2]	8 (28)	29 (39)	
**Clinical characteristics**			
Disease duration before LP (months)[1]	0.8 (0-11.5)	0.6 (0-12.7)	0.792[3]
Follow-up duration (years)[1]	5.8 (0.8-12.3)	10.0 (2.0-16.1)	**<0.001**[3]
Disease duration until 2nd relapse (years)[1]	NA	1.6 (0.2-7.4)	NA

Legend:

Data are shown as [1]median (5th-95th percentile) or [2]n (%). Group comparison were performed by [3]Mann-Whitney U test or [4]Pearson Chi-Quadrat test. P-values <0.05 are marked bold.

Abbreviations: DIS, dissemination in space; DIT, dissemination in time; LP, lumbar puncture; MRI, magnetic resonance imaging; NA, not appropriate

Table S4: Correlation of various cerebrospinal fluid findings

		CSF TP (mg/l)	Q_{alb}	IF IgG Reiber (%)	IF IgG Auer&Hegen (%)	IF IgA Reiber (%)	IF IgA Auer&Hegen (%)	IF IgM Reiber (%)	IF IgM Auer&Hegen (%)	Age[1] (years)	Disease duration[1] (years)
	r	0.220	0.111	0.427	0.459	0.119	0.137	0.237	0.240	-0.282	-0.135
**WBC (/µl)**	p-value	**<0.001**	**0.011**	**<0.001**	**<0.001**	**0.007**	**0.002**	**<0.001**	**<0.001**	**<0.001**	**0.002**
	n	540	527	527	527	517	517	527	527	541	540
	r		0.794	-0.046	0.054	-0.128	-0.025	-0.200	-0.185	0.165	0.158
**CSF TP (mg/l)**	p-value		**<0.001**	0.289	0.215	**0.004**	0.569	**<0.001**	**<0.001**	**<0.001**	**<0.001**
	n		526	526	526	516	516	526	526	540	539
	r			-0.323	-0.208	-0.160	-0.048	-0.260	-0.247	0.224	0.153
**Q_{alb}**	p-value			**<0.001**	**<0.001**	**<0.001**	0.272	**<0.001**	**<0.001**	**<0.001**	**<0.001**
	n			527	527	517	517	527	527	527	526
	r				0.991	0.341	0.304	0.390	0.385	-0.063	0.028
**IF IgG Reiber (%)**	p-value				**<0.001**	**<0.001**	**<0.001**	**<0.001**	**<0.001**	0.146	0.528
	n				527	517	517	527	527	527	526
	r					0.328	0.306	0.367	0.364	-0.038	0.052
**IF IgG Auer&Hegen (%)**	p-value					**<0.001**	**<0.001**	**<0.001**	**<0.001**	0.379	0.231
	n					517	517	527	527	527	526
	r						0.985	0.530	0.523	-0.126	-0.073
**IF IgA Reiber (%)**	p-value						**<0.001**	**<0.001**	**<0.001**	**0.004**	0.098
	n						517	517	517	517	516
	r							0.499	0.500	-0.107	-0.047
**IF IgA Auer&Hegen (%)**	p-value							**<0.001**	**<0.001**	**0.015**	0.289
	n							517	517	517	516
	r								0.997	-0.156	-0.091
**IF IgM Reiber (%)**	p-value								**<0.001**	**<0.001**	**0.037**
	n								527	527	526

**IF IgM $_{Auer\&Hegen}$ (%)**	r									-0.158	-0.079
	p-value									**<0.001**	0.07
	n									527	526

<u>Legend</u>:

[1] Patients' age and disease duration was determined at the time of lumbar puncture. P-values <0.05 are marked bold.

Abbreviations: CSF, cerebrospinal fluid; IF, intrathecal fraction; Ig, immunoglobulin; n, number of patients; Q_{alb}, CSF/ serum albumin quotient; r, correlation coefficient; TP, total protein; WBC, white blood cell

Table S5: Linear regression analysis to identify predictors of CSF white blood cells

**Variable**	**Coefficient**	**SE**	**95% CI**		**P value**
Sex[1]	-0.274	0.098	-0.466	-0.081	**0.005**
Age (years)	-0.704	0.167	-1.032	-0.375	**<0.001**
Disease duration (years)	-0.045	0.030	-0.104	0.014	0.138
Diagnosis[2]					
CIS	0.477	0.233	0.019	0.934	**0.041**
McDonald MS	0.682	0.204	0.281	1.083	**0.001**
RRMS	0.821	0.183	0.462	1.179	**<0.001**
SPMS	0.502	0.286	-0.059	1.063	0.079
Constant	3.627	0.653	2.344	4.909	<0.001

<u>Legend</u>:

The dependent variable WBC count (/µl) and the independent variables age (determined at the time of lumbar puncture) and disease duration (time between symptom onset and lumbar puncture) were log-transformed to achieve normal distribution.

Reference categories were [1] male sex and [2] PPMS. P-values <0.05 are marked bold.

Abbreviations: CI, confidence interval; CIS, clinically isolated syndrome; CSF, cerebrospinal fluid; MS, multiple sclerosis; PPMS, primary progressive multiple sclerosis; RRMS, relapsing-remitting multiple sclerosis; SE, standard error; WBC, white blood cell

Table S6: Linear regression analysis to identify predictors of Q_{alb}

Variable	Coefficient	SE	95% CI		P value
Sex[1]	-0.251	0.038	-0.325	-0.176	**<0.001**
Age (years)	0.283	0.065	0.156	0.411	**<0.001**
Disease duration (years)	0.015	0.012	-0.008	0.038	0.192
Diagnosis[2]					
CIS	-0.148	0.129	-0.402	0.106	0.253
McDonald MS	-0.040	0.121	-0.278	0.198	0.740
RRMS	-0.039	0.109	-0.254	0.176	0.722
PPMS	-0.191	0.120	-0.426	0.044	0.111
Constant	0.845	0.265	0.324	1.366	0.002

Legend:

The dependent variable Q_{alb} and the independent variables age (determined at the time of lumbar puncture) and disease duration (time between symptom onset and lumbar puncture) were log-transformed to achieve normal distribution.

Reference categories were [1] male sex and [2] SPMS. P-values <0.05 are marked bold.

Abbreviations: CI, confidence interval; CIS, clinically isolated syndrome; MS, multiple sclerosis; PPMS, primary progressive multiple sclerosis; Q_{alb}, CSF/ serum albumin quotient; RRMS, relapsing-remitting multiple sclerosis; SE, standard error

Table S7: Linear regression analysis to identify predictors of CSF total protein

Variable	Coefficient	SE	95% CI		P value
Sex[1]	-0.191	0.030	-0.251	-0.132	**<0.001**
Age (years)	0.141	0.052	0.039	0.244	**0.007**
Disease duration (years)	0.009	0.009	-0.01	0.027	0.362
Diagnosis[2]					
CIS	-0.134	0.098	-0.328	0.059	0.172
McDonald MS	-0.075	0.091	-0.254	0.104	0.411
RRMS	-0.046	0.080	-0.204	0.112	0.565
PPMS	-0.137	0.089	-0.311	0.038	0.126
Constant	3.396	0.211	2.982	3.810	<0.001

Legend:

The dependent variable CSF total protein and the independent variables age (determined at the time of lumbar puncture) and disease duration (time between symptom onset and lumbar puncture) were log-transformed to achieve normal distribution.

Reference categories were [1] male sex and [2] SPMS. P-values <0.05 are marked bold.

Abbreviations: CI, confidence interval; CIS, clinically isolated syndrome; MS, multiple sclerosis; PPMS, primary progressive multiple sclerosis; RRMS, relapsing-remitting multiple sclerosis; SE, standard error

Table S8: Linear regression analysis to identify predictors of intrathecal IgG synthesis

Variable	Coefficient	SE	95% CI		P value
Sex[1]	7.931	3.426	1.2	14.662	**0.021**
Age (years)	-10.07	5.846	-21.556	1.416	0.086
Disease duration (years)	0.042	1.054	-2.029	2.113	0.968
Diagnosis[2]					
CIS	-36.717	11.678	-59.659	-13.775	**0.002**
McDonald MS	-22.255	10.932	-43.733	-0.777	**0.042**
RRMS	-21.908	9.86	-41.279	-2.537	**0.027**
PPMS	-21.065	10.803	-42.289	0.159	0.052
Constant	67.494	23.946	20.448	114.54	0.005

Legend:

The dependent variable was the intrathecal fraction (%) of IgG as determined by the Auer & Hegen formula. The independent variables age (determined at the time of lumbar puncture) and disease duration (time between symptom onset and lumbar puncture) were log-transformed to achieve normal distribution. Reference categories were [1] male sex and [2] SPMS. P-values <0.05 are marked bold.

Abbreviations: CI, confidence interval; CIS, clinically isolated syndrome; IgG, immunoglobulin G; MS, multiple sclerosis; PPMS, primary progressive multiple sclerosis; RRMS, relapsing-remitting multiple sclerosis; SE, standard error

Table S9: Binary logistic regression analysis to identify predictors of CSF-restricted oligoclonal bands

Variable	Odd' s ratio	95% CI for OR		P value
Sex[1]	1.993	0.710	5.593	0.19
Age (years)	1.018	0.961	1.078	0.552
Disease duration (years)	2.315	0.185	28.932	0.515
Brain MRI activity[2]				
DIS	3.479	0.860	14.071	0.080
DIT	1.806	0.348	9.376	0.482
DIS & DIT	9.611	2.482	37.212	**0.001**
Constant	3.741	0.550	25.474	0.178

Legend:

The dependent variable was OCB status (either positive or negative). Reference categories were [1] male sex and [2] no DIS & DIT. P-values <0.05 are marked bold. 95% CI is given for the Odd's ratio.

Abbreviations: CI, confidence interval; CSF, cerebrospinal fluid; DIS, dissemination in space; DIT, dissemination In time; SE, standard error; OCB, oligoclonal bands

Table S10: Binary logistic regression analysis to identify predictors of conversion to CDMS

Variable	Odds ratio	95% CI		P value
Sex[1]	0.962	0.306	3.025	0.947
Age (years)	1.010	0.947	1.077	0.761
Follow-up duration (years)	1.246	1.096	1.416	**0.001**
Monocytes/ macrophages[2]	7.314	1.401	38.197	**0.018**
CSF pleocytosis[3]	2.872	1.005	8.204	**0.049**
Constant	0.962	0.306	3.025	0.142

Legend:

The dependent variable was occurrence of second relapse during follow-up (i.e. relapse or non-relapse). Reference categories were [1] male sex and [2] no monocytes/ macrophages (in CSF cytology) and [3] no CSF pleocytosis (i.e. WBC count <5/μl). P-values <0.05 are marked bold. 95% CI is given for the Odd's ratio.

Abbreviations: CI, confidence interval; CMDS, clinically definite multiple sclerosis; CSF, cerebrospinal fluid; WBC, white blood cell.

Table S11: Quantitative intrathecal immunoglobulin synthesis according to oligoclonal band status

	**OCB negative**		**OCB positive**	
	n		n	
Intrathecal IgG synthesis				
IF_{Reiber} >0	26	0 (0)	494	353 (71)
$IF_{Auer\&Hegen}$ >0	26	0 (0)	494	338 (68)
IgG index >0.7	26	1 (4)	494	356 (72)
Intrathecal IgA synthesis				
IF_{Reiber} >0	26	1 (4)	484	117 (24)
$IF_{Auer\&Hegen}$ >0	26	1 (4)	484	73 (15)
Intrathecal IgM synthesis				
IF_{Reiber} >0	26	1 (4)	494	172 (35)
$IF_{Auer\&Hegen}$ >0	26	1 (4)	494	111 (22)

Legend:

Data are shown as n (%). *Abbreviations*: IF, intrathecal fraction; Ig, immunoglobulin; OCB, oligoclonal bands
